# Professional Identity Formation in Clinical Medical Education: A Multicenter Narrative Study of Contextual Mediation and Reflective Meaning-Making

**DOI:** 10.5334/pme.2658

**Published:** 2026-11-04

**Authors:** Selçuk Akturan, Merve Saraçoğlu, Elif Deniz Şafak, Şükrü Keleş, Bilge Delibalta, Gökhan Kumlu, İnanç Sümbüloğlu, Beril Müge Güçlü, Hanife Suay Ayvat, Mehmet Hatip Coşkun, Mert Kaan Güven, Yasemin Güner, Aslı Boz, Türkkan Öztürk Kaygusuz, Selma Aydın, Mehmet Ali Gülpınar, Timothy Fredric Murphy, Ara Tekian

**Affiliations:** 1 Department of Medical Education, Faculty of Medicine, Karadeniz Technical University, Trabzon, Turkey; 2 Department of Medical Education, Faculty of Medicine, Recep Tayyip Erdoğan University, Rize, Turkey; 3 Department of Family Medicine, Faculty of Medicine, Erciyes University, Kayseri, Turkey; 4 Department of Medical History and Ethics, Faculty of Medicine, Karadeniz Technical University, Trabzon, Turkey; 5 Department of Educational Sciences, Faculty of Education, Sinop University, Sinop, Turkey; 6 Department of Medical Education, Institute of Health Science, Marmara University, Istanbul, Turkey; 7 Faculty of Medicine, Ege University, Izmir, Turkey; 8 Faculty of Medicine, Fırat University, Elazığ, Turkey; 9 Faculty of Medicine, Dicle University, Diyarbakır, Turkey; 10 Department of Medical Education, Institute of Health Sciences, Acibadem Mehmet Ali Aydinlar University, Istanbul, Turkey; 11 Department of Medical Education, Faculty of Medicine, Çukurova University, Adana, Turkey; 12 Department of Infectious Diseases, Faculty of Medicine, Fırat University, Elazığ, Turkey; 13 Department of Medical Education, Faculty of Medicine, Başkent University, Ankara, Turkey; 14 Department of Medical Education, Faculty of Medicine, Marmara University, Istanbul, Turkey; 15 Department of Medical Education, University of Illinois Chicago, Chicago, USA

## Abstract

**Introduction:**

Understanding how physicians interpret their societal and institutional contexts remains crucial for addressing the complexities of professional identity. This study explores professional identity formation (PIF) in clinical medical education through learners’ narratives and the perspectives of their role models.

**Methods:**

Using a multi-center qualitative narrative design, data were collected from clinical-year medical students across accredited state and private medical faculties representing diverse geographical regions. Reflective narrative writing and in-depth interviews were analyzed through a staged contextual qualitative approach.

**Results:**

Findings indicate that PIF is shaped through the dynamic interplay between clinical environments, societal and systemic structures, role modelling, and learners’ reflective engagement. Narratives framed professional identity not only in terms of technical competence but also through relational, ethical, and humanistic dimensions. Contextual analysis revealed that tensions between service demands and educational engagement, hierarchy and learner agency, and technical efficiency and humanistic care significantly influenced identity development. Reflection and narrative meaning-making emerged as mediating processes through which learners interpreted and internalized these experiences.

**Discussion:**

Drawing on a socio-cultural paradigm informed by activity theory, the study proposes a conceptual framework positioning PIF as a relational and contextually mediated construction shaped by both planned and implicit learning processes. The findings underscore the need for medical education programs to intentionally integrate reflective, narrative, and context-sensitive approaches to support sustainable professional identity development.

## 1. Introduction

Professional identity formation (PIF) is a fundamental process in medical education through which learners progressively integrate the values, responsibilities, norms, and ways of thinking of the medical profession into their evolving sense of self as physicians [1,2]. Although closely related, PIF is conceptually distinct from professionalism. Professionalism primarily refers to the values, behaviours, and standards expected of physicians, whereas PIF concerns the developmental process through which learners interpret, negotiate, internalize, and continuously reconstruct these expectations as part of becoming physicians [3]. Accordingly, contemporary medical education increasingly recognizes that preparing competent physicians requires not only teaching professional behaviours but also supporting learners in developing a professional identity capable of sustaining ethical, reflective, and patient-centred practice [4,5].

PIF is now widely conceptualized as a dynamic, socially negotiated, and context-dependent developmental process rather than the acquisition of professional competencies alone. Contemporary perspectives emphasize that professional identity evolves through learners’ active engagement with authentic clinical experiences, participation in communities of practice, relationships with others, and the ongoing interpretation of personal and professional experiences within specific sociocultural contexts. Recent conceptual models further highlight learner agency, critical engagement with professional norms, and the interconnected processes of being, becoming, and belonging as central mechanisms through which professional identities develop [6,7,8]. During clinical training, learners participate in professional communities where they encounter the formal curriculum, informal learning experiences, and the hidden curriculum. Together, these educational experiences shape how learners interpret professional values, negotiate expectations, and envision their future professional selves. Within these environments, role models become particularly influential because they embody the values, behaviours, and professional norms that learners observe in everyday practice. Positive role models may reinforce learners’ sense of belonging, professional commitment, and identity development, whereas discrepancies between formally taught values and observed clinical practice may generate uncertainty, conflict, or identity dissonance [9,10]. Importantly, role modelling also represents one of the principal ways through which the hidden curriculum becomes visible to learners, as organizational values, institutional norms, and workplace cultures are often communicated implicitly through everyday interactions rather than formal instruction.

To better understand these contextual influences, this study adopts third-generation Activity Theory as its primary theoretical framework. Activity Theory conceptualizes learning and identity formation as emerging through participation in interconnected activity systems, where individuals, communities, institutional rules, shared objectives, mediating tools, and divisions of labour continuously interact [11]. The first generation of Activity Theory, based on Vygotsky’s work, focused on individuals’ mediated actions and the role of cultural artifacts in shaping human learning and development. The second generation, further developed by Engeström, expanded this perspective by conceptualizing activity as a collective activity system and emphasizing its structure, social interactions, contradictions, and development over time [11]. Building on these perspectives, third-generation Activity Theory examines the interactions and relationships between multiple activity systems, highlighting how tensions, contradictions, and collaborations across systems shape human activity and development [12]. This perspective is particularly well suited to examining PIF because it moves beyond individual learners or isolated interpersonal interactions to explain how broader educational, organizational, and healthcare structures influence identity development. Within this framework, role modelling represents an interpersonal mechanism through which learners encounter and interpret the values, expectations, and contradictions embedded within these interacting systems.

Although an increasing body of research acknowledges the contextual nature of PIF, much of the qualitative literature has been conducted within single institutions or relatively homogeneous educational settings, limiting understanding of how different organizational and clinical contexts influence identity development [13,14,15]. Consequently, there remains limited evidence regarding how PIF is shaped across multiple clinical learning environments operating within a shared medical education and healthcare system. A multi-institutional qualitative approach provides an opportunity to identify both common and context-specific mechanisms influencing PIF and to extend current understanding of how organizational environments, clinical cultures, and contextual conditions contribute to professional identity development.

Understanding these contextual mechanisms has important implications beyond describing learners’ experiences. Identifying how organizational structures, clinical environments, hidden curricula, and role modelling collectively influence PIF can inform curriculum design, faculty development, and the creation of learning environments that intentionally support identity development alongside professional competence.

Accordingly, the aim of this study was to explore how clinical medical students and the physicians they identified as role models interpreted the influence of clinical, educational, and organizational contexts on PIF. Guided by third-generation Activity Theory, this study addressed the following research question:

How do clinical, educational, and organizational contexts shape PIF through the experiences of clinical medical students and the physicians they identify as role models?

## 2. Materials and Methods

### 2.1. Study Design

This multicenter qualitative study employed a narrative approach, informed by third-generation Activity Theory, to explore how clinical, educational, and organizational contexts shape PIF among clinical medical students and the physicians they identified as role models. A qualitative design was considered appropriate because the study aimed to understand how participants interpreted and constructed meaning from their experiences within authentic clinical learning environments.

Narrative data were used because PIF develops through participants’ interpretations of significant clinical experiences. Rather than treating narratives solely as descriptions of events, the study examined how learners and role models made sense of influential clinical encounters, organizational cultures, and tensions between educational ideals and everyday clinical practice.

Third-generation Activity Theory provided the conceptual framework for understanding these experiences within interacting educational and healthcare systems. This perspective informed both the development of the data collection instruments and the interpretation of contextual influences during analysis.

To obtain a comprehensive understanding of participants’ experiences, two complementary qualitative data sources were used: reflective narrative writings and semi-structured in-depth interviews. Reflective narratives enabled participants to identify significant experiences, whereas interviews provided opportunities to further explore their meanings, contextual influences, and identity-related interpretations. These complementary data sources were analyzed separately and subsequently integrated through the contextual analysis framework to develop a comprehensive understanding of professional identity formation across diverse clinical learning environments.

### 2.2. Study Context

This The Scientific and Technological Research Council of Türkiye (TÜBİTAK)-funded multicenter qualitative study was conducted in nine accredited medical schools located across Türkiye’s seven geographical regions. Participating institutions were selected from medical schools accredited by the Association for Evaluation and Accreditation of Medical Education Programs (TEPDAD) to ensure comparable educational quality while providing variation in institutional and clinical contexts. Of the nine selected medical schools, seven were public institutions, with one institution representing each of Türkiye’s seven geographical regions. The remaining two were private medical schools that met the predefined eligibility criteria; although private medical schools existed in other regions, they did not meet the eligibility criteria during the study period. The participating medical schools differed in their geographical location, institutional history, organizational structure, patient populations, and clinical service environments. These differences provided diverse clinical learning contexts in which professional identity formation could be explored and enabled examination of how organizational cultures, educational environments, and clinical practices influenced learners’ professional identity development across multiple institutional settings. From the perspective of third-generation Activity Theory, these institutional differences represented varied educational and clinical activity systems within which professional identity formation occurred.

A summary of the characteristics of the nine participating institutions is presented in Table 1.

**Table 1:** Characteristics of participating medical schools and overview of data collection across institutions.


SCHOOL OF MEDICINE	INSTITUTION TYPE	GEOGRAPHICAL REGION	NUMBER OF OBTAINED NWS	NUMBER OF IN-DEPTH INTERVIEWS WITH STUDENTS	REVEALED ROLE MODELS	INTERVIEWED RMS

Acıbadem University Faculty of Medicine	Private	Marmara	9	9	7	4

Başkent University Faculty of Medicine	Private	Central Anatolian	7	6	5	3

Çukurova University Faculty of Medicine	Public	Meditarrenean	9	9	8	8

Dicle University Faculty of Medicine	Public	Southeastern Anatolia	9	9	6	4

Ege University Faculty of Medicine	Public	Agean	9	9	9	8

Erciyes University Faculty of Medicine	Public	Central Anatolian	9	9	9	9

Fırat University Faculty of Medicine	Public	Eastern Anatolia	6	6	5	5

Karadeniz University Faculty of Medicine	Public	Blacksea	9	9	6	5

Marmara University Faculty of Medicine	Public	Marmara	8	8	6	4

**Total**			**75** **92.6%**	**74** **98.7%**	61*	**50** **82.0%**


*7 of the 61 role models determined could not be reached.

### 2.3. Participants and Sampling

A criterion-based purposive sampling strategy was used to select participating institutions, while eligible participants were recruited on a voluntary basis through the administrations of the participating medical schools. To maximize contextual diversity, the study included public and private medical schools from Türkiye’s seven geographical regions. Participating institutions were selected according to predefined criteria: (1) accreditation by the TEPDAD; (2) for public medical schools, a minimum institutional history of 30 years; and (3) for private medical schools, a minimum institutional history of 10 years. Public medical schools meeting these criteria were additionally selected based on institutional feasibility. Following institutional approvals, researchers from each participating institution (and, where appropriate, two researchers) were involved in institutional coordination, participant recruitment, data collection, and contextual interpretation. Their familiarity with local educational and clinical environments enhanced contextual sensitivity, facilitated participant engagement, and ensured methodological consistency across participating institutions. Written informed consent was obtained from all participants.

Eligible participants were medical students in the clinical phase of training (Years 4–6 of the six-year Turkish medical program). In Years 4 and 5, students undertake clinical clerkships in hospital and primary care settings under the supervision of faculty members, progressively assuming greater responsibility for patient care while continuing structured educational activities. Year 6 constitutes the internship year, during which students work as interns with greater clinical responsibilities and participate more directly in patient care under supervision. Students who had completed at least six months of clinical clerkship training were eligible, ensuring sufficient clinical exposure to reflect on meaningful experiences related to professional identity formation. Students who voluntarily agreed to participate first completed reflective narrative writings and were subsequently invited to participate in semi-structured interviews. Role models were identified inductively through students’ reflective narratives and interviews. Physicians who were repeatedly described as influential in students’ professional identity development were identified as potential role models and invited to participate in individual interviews exploring their perspectives on professional identity formation and clinical education. In total, 61 physicians were identified as potential role models, of whom 50 (82.0%) agreed to participate in the interviews. Participant characteristics and distribution across institutions are presented in Table 1.

### 2.4. Data Collection

Data were collected sequentially using two complementary qualitative methods: **reflective narrative writing** and **semi-structured in-depth interviews**. This combination enabled both the identification of personally meaningful clinical experiences and a deeper exploration of the contextual factors shaping PIF.

#### 2.4.1. Reflective Narrative Writing

Reflective narrative writing is a tool that allows individuals to analyze lived experiences and gain deeper insights into daily professional practices that might otherwise go unnoticed. Reflective narrative writing provides an opportunity for medical professionals to process and learn from their experiences effectively [16].

Clinical medical students first completed reflective narrative writings using a structured reflective writing guide adapted from Hampton’s Reflective Writing Guide (2010) [17]. The guide was reviewed by experts in medical education and piloted with ten volunteer students to evaluate its clarity, relevance, and usability. Minor revisions were subsequently made to strengthen the reflection and future planning sections.

Before narrative data collection, all students who voluntarily agreed to participate in the study attended a standardized online three-hour interactive reflective writing workshop conducted at their respective medical schools. A total of nine workshops were delivered by six members of the research team. Prior to implementation, the facilitators met online to establish a common instructional framework, ensuring consistency in the learning objectives, content, and delivery of the workshops across all participating institutions. The workshops introduced the concepts of professional identity formation and reflective writing, familiarized participants with the reflective writing guide, and included guided writing exercises with feedback to promote a shared understanding of reflective narrative writing before data collection.

Students were then asked to write narratives describing significant clinical experiences that had influenced their professional identity formation, including interactions with role models, challenging clinical situations, and experiences that shaped their understanding of becoming a physician. These narratives served as the primary source for identifying meaningful experiences and informed the subsequent interview phase.

#### 2.4.2. Semi-Structured In-Depth Interviews

Semi-structured in-depth interviews aim to gather detailed insights into participants’ perspectives, experiences, emotions, and meanings related to a specific theme [18].

Semi-structured interviews were subsequently conducted with selected students to further explore the experiences described in their narratives, clarify emerging meanings, and examine the contextual factors influencing professional identity formation. Physicians identified by students as influential role models were also invited to participate in individual interviews.

Interview guides were developed in accordance with the study aims and the conceptual framework provided by third-generation Activity Theory, with particular attention given to participants’ experiences of clinical learning environments, organizational influences, role modelling, and professional identity formation. The interview guide is provided in Supplementary Material 1.

### 2.5. Data Analysis

All interviews were audio-recorded and transcribed verbatim by two researchers using transcription software. Each transcript was subsequently verified through repeated listening to the original recordings, and any discrepancies or omissions were resolved through discussion until consensus was reached. As all interviews were conducted in Turkish, data analysis was performed in the original language to preserve contextual meanings. Quotations included in the manuscript were translated into English by the research team and reviewed to ensure conceptual and contextual equivalence.

Reflective narratives and interview transcripts were initially analyzed separately using the Three-Stage Contextual Reading and Analysis Framework developed by Gülpınar (2024) [19], a qualitative analytical framework specifically designed to identify, interpret, and report contextual influences embedded within narrative data. Separate analyses enabled each dataset to retain its distinctive contextual characteristics before findings were integrated during the final interpretive stage.

In **Phase I (Initial Contextual Reading)**, two researchers (SA and MS) independently conducted repeated readings and open coding to identify initial codes, contextual dimensions, and emerging categories. Coding discrepancies were discussed until consensus was achieved, with consultation from a third researcher (MAG) when necessary.

In **Phase II (Conceptual Framing)**, preliminary themes and relationships among contextual factors were iteratively synthesized into an initial analytical framework. This phase involved refining the analytical focus and organizing emerging patterns to facilitate deeper interpretation of professional identity formation across diverse clinical contexts.

In **Phase II (Conceptual Framing)**, preliminary themes and relationships among contextual factors were iteratively synthesized into an initial analytical framework. The analytical focus was progressively refined, and emerging patterns were organized to facilitate deeper interpretation of professional identity formation across diverse clinical contexts.

In **Phase III (Advanced Contextual Interpretation)**, findings from the reflective narratives and interviews were interpreted together using the refined analytical framework. Themes and subthemes were compared across participant groups and institutions, allowing confirmation, refinement, or expansion of the emerging conceptual categories. Throughout this process, third-generation Activity Theory served as a sensitizing framework for interpreting how interactions among educational, organizational, and clinical contexts influenced professional identity formation.

The final themes and conceptual framework were developed through iterative discussions among the research team until analytical consensus was achieved. This iterative analytical process, together with investigator triangulation involving multiple researchers throughout coding, interpretation, and framework development, enhanced the credibility and trustworthiness of the findings.

### 2.6. Research Team and Reflexivity

The research team was interdisciplinary and comprised family physicians, a nurse with doctoral training in medical education, a clinical psychologist, and an assessment specialist, bringing expertise in qualitative research, professional identity formation, curriculum development, simulation-based education, and clinical practice. The principal investigator (SA) had approximately ten years of clinical experience across diverse healthcare settings and academic expertise in medical education. The project advisor (MAG) contributed theoretical expertise in contextuality, complexity, and narrative approaches.

Throughout the study, the research team engaged in regular reflexive discussions to critically examine how their disciplinary backgrounds, clinical experiences, and prior assumptions might influence data collection, coding, interpretation, and framework development. These discussions supported reflexivity throughout the research process and contributed to the methodological rigor and credibility of the findings.

### 2.7. Trustworthiness

The trustworthiness of the study was established in accordance with the criteria proposed by Lincoln and Guba, including credibility, dependability, confirmability, and transferability. Credibility was enhanced through investigator triangulation, iterative discussions among researchers during coding and interpretation, and the use of complementary qualitative data sources (reflective narratives and semi-structured interviews). Dependability and confirmability were supported through systematic documentation of analytical decisions, iterative refinement of themes, and consensus meetings throughout the analytical process. Transferability was strengthened by the multicenter design, inclusion of diverse institutional contexts, and detailed description of the study context, participants, and analytical procedures, enabling readers to determine the applicability of the findings to other educational settings.

### 2.8. Ethical Approval

Ethical approval for the study was obtained from the Scientific Research Ethics Committee of Karadeniz Technical University Faculty of Medicine (Approval No: 2021/180; Date: June 23, 2021). Institutional permissions were obtained from all participating medical schools prior to data collection. All participants received written information about the study and provided informed consent before participation. Participation was voluntary, and participants were informed that they could withdraw from the study at any time without consequence. To ensure confidentiality, all identifying information was removed during transcription and analysis, and pseudonyms were used when reporting qualitative findings.

## 3. Results

The findings indicate that PIF was shaped by the interplay of clinical, educational, and organizational contexts across diverse clinical learning environments (Table 2). Rather than being influenced by contextual factors directly, professional identity developed through learners’ active interpretation of authentic clinical experiences, role-model behaviours, and organizational tensions. This interpretive process emerged as a central mechanism through which learners assigned personal and professional meaning to their experiences and incorporated them into their developing professional identities. The findings are presented under three analytical domains: clinical, educational, and organizational contexts.

**Table 2:** Analytical domains derived from the research questions and corresponding themes.


ANALYTICAL DOMAIN	THEME	SUBTHEMES	ANALYTICAL DESCRIPTION

**Framing Professional Identity in Clinical Narratives**	**Professional identity as moral and relational negotiation**	Balancing ethics, systemic constraints, and personal conscience Responsibility and accountability in clinical decision-making Humanistic and relational dimensions of medical practice	Professional identity is narrated as an ongoing negotiation between personal values, ethical responsibilities, and contextual demands encountered in clinical practice.

	**Role modeling as identity anchoring**	Humility, consistency, and ethical conduct Internalization of observed professional behaviors	Role models function as embodied reference points through which learners interpret, evaluate, and construct their emerging professional identities.

**Contextual Forces Shaping Professional Identity Formation**	**Clinical and institutional contexts of professional learning**	Healthcare environment (workload, hierarchy, collaboration) Clinical learning opportunities and informal learning Embodied approaches, attitudes, and professional rituals	Institutional climate, participation opportunities, and hierarchical cultures shape how learners experience, interpret, and enact professional roles.

	**Societal, cultural, and systemic influences**	Societal expectations and perceptions of physicians Systemic constraints within healthcare and medical education	Broader societal narratives and system-level pressures influence learners’ perceptions of professional responsibility, authority, and belonging.

**Reflective and Transformative Engagement in Clinical Practice**		Self-reflection and learning from experience Critical thinking and clinical reasoning Emotional and cognitive engagement	Clinical experiences are processed through reflection, reasoning, and emotional engagement, shaping how learners consolidate, question, and reconstruct their developing professional identities.


Quotations are presented using a standardized coding system in which the first letter denotes the participating medical school, the number represents the participant identification number assigned by the responsible researcher, and the final abbreviation identifies the participant type and data source (RY = reflective writing, DG = in-depth interview, and RM = role model interview.

### 3.1. Framing Professional Identity in Clinical Narratives

Across institutions, participants described PIF as a dynamic process of negotiating competing clinical, ethical, organizational, and personal responsibilities, rather than the acquisition of professional competencies alone. Professional identity emerged through participants’ attempts to reconcile patient needs, healthcare system demands, ethical principles, and personal values while engaging in everyday clinical practice.

One student illustrated this multidimensional understanding of professional responsibility:

*“I believe that physicians are the key stakeholders in keeping the healthcare system running in Türkiye. We have to think about the things the system fails to address or even consider… When prescribing a treatment*, *we must consider the state’s budget, the patient’s circumstances, medical ethics, and our own conscience.” (ER3AY2)*

Role models similarly described professional identity as extending beyond technical expertise to include responsibility, accountability, and commitment to solving patients’ problems despite contextual constraints.

*“We should never avoid any patient or problem just because it seems too big or complex… our fundamental mission should be to fully assess the patient, thoroughly understand the issue, and develop our professional skills to find the most suitable solution for them.” (E8-Role Model)*

Professional identity was also constructed through observation of everyday clinical practice, where learners interpreted the behaviours of experienced physicians as models for their own professional development. Rather than simply adopting observed behaviours, participants actively interpreted these experiences and integrated them into their evolving understanding of what it means to become a physician.

*“…I felt the importance of staying attentive, not missing details, and understanding what is expected from a physician. I started observing myself more carefully. This experience deeply affected how I perceived my mentor’s professional identity.” (A7AY)*

Participants frequently contrasted technical competence with humanistic practice, suggesting that clinical expertise alone was insufficient for defining professional identity. Human-centred interactions, emotional presence, and compassionate communication consistently emerged as distinguishing characteristics of exemplary physicians.

*“…Some surgeons approached the body as if it were a machine. They would tighten loose screws and replace broken parts. But Professor X never forgot that the body was a human being.” (M4AY)*

Similarly, emotionally meaningful encounters reinforced participants’ understanding that professionalism involved relational engagement as much as scientific competence.

*“One of the most impactful moments during my training was when I saw a physician comfort a patient before a painful procedure… Watching that interaction made me realize that being a good doctor isn’t just about medical knowledge—it’s also about how we make patients feel.” (A7AY)*

Role models themselves framed professional identity in terms of enduring personal values rather than professional status. Humility, integrity, and ethical consistency were repeatedly described as essential characteristics of becoming and remaining a physician.

*“I have my own rules and principles… I don’t accept gifts from patients.”* (D1RM)

Across narratives, professional identity was therefore constructed as a relational, ethically grounded, and contextually negotiated process, shaped not only by formal professional expectations but also by learners’ interpretations of authentic clinical experiences and the everyday practices of respected role models.

### 3.2. Contextual Forces Shaping Professional Identity Formation

Participants described clinical environments, hierarchical structures, and broader systemic conditions as shaping how professional identity developed in practice.

#### 3.2.1. Clinical learning environments

Clinical learning environments emerged as one of the most influential contextual forces shaping professional identity formation. Across institutions, participants consistently described identity development as being strongly influenced by how they were included in everyday clinical practice, rather than simply by exposure to clinical knowledge. Learning environments that promoted participation, psychological safety, and supportive supervision enabled students to move from peripheral observation toward active engagement within the clinical team. These experiences fostered a sense of professional belonging and reinforced learners’ perceptions of themselves as future physicians.

One student described how supportive interactions transformed feelings of uncertainty into a sense of legitimate participation:

*“Before the ward round, I was quite anxious about how things would unfold. But as the interaction progressed, I realized my worries were unnecessary. The way the team communicated and involved me made me feel that I truly belonged there.” (M4AY)*

Similarly, opportunities to participate directly in patient care were described as important milestones in identity development. Rather than merely observing clinical encounters, students reported that being recognized as contributing members of the healthcare team strengthened both their confidence and their developing professional identity.

*“My professor introduced me to the patient as part of the team and invited me to participate in the case. At that moment, I no longer felt like an observing student in the hospital, but like a real member of the clinical team.” (A5AY)*

Role models also emphasized that meaningful participation rarely occurred automatically but required deliberate educational support despite organizational constraints. They described intentionally creating learning opportunities that enabled students to progressively assume greater clinical responsibility, even within busy service-oriented environments.

*“I encourage my students to see as many patients as possible… This requires extra effort—staying for night shifts, engaging with residents in surgeries, and seizing opportunities to practice skills …” (E8RM)*

Conversely, fragmented communication, limited supervision, and unclear expectations often disrupted participation and generated uncertainty regarding learners’ roles within the clinical environment. Rather than facilitating PIF, these experiences reinforced students’ peripheral status and reduced opportunities for active engagement.

*“While I was observing the case, the physician suddenly stopped what he was doing and left the room without saying anything. I stayed there, unsure whether I should follow or wait, trying to understand what had just happened.” (B5AY)*

Across narratives, clinical learning environments influenced professional identity formation not simply through the transfer of clinical knowledge but through the extent to which learners were recognized, supported, and entrusted with meaningful participation in authentic clinical practice. Supportive supervision, inclusive team interactions, and structured participation emerged as key mechanisms through which clinical environments facilitated learners’ transition toward professional membership.

#### 3.2.2. Hierarchy and leadership

Hierarchy emerged as a pervasive feature of clinical learning environments, shaping not only how work was organized but also how learners participated, communicated, and positioned themselves within professional communities. Participants described hierarchy as having a dual influence on PIF. On one hand, it provided organizational structure and clarified professional responsibilities; on the other, rigid hierarchical relationships often constrained questioning, limited participation, and reinforced passive learning.

Role models consistently distinguished effective leadership from individual authority, emphasizing collaboration, shared responsibility, and collective learning rather than personal expertise or status. Leadership was described as creating conditions in which every team member could contribute meaningfully to patient care and learning.

*“Doctors do not have heroes; teams do.” (K2RM)*

*“Everyone should feel that they are an essential part of the team and that the system cannot function without their contribution.” (Ç4RM)*

In contrast, students frequently described hierarchical norms as influencing their willingness to participate in clinical discussions. Rather than encouraging dialogue, some clinical environments implicitly communicated that questioning senior physicians might be interpreted as challenging authority. Consequently, learners often prioritized compliance over inquiry, limiting opportunities for critical engagement and professional growth.

*“In highly hierarchical settings, I sometimes felt that following instructions was expected, while asking questions could be interpreted as challenging authority.” (M4AY)*

Several narratives suggested that these experiences extended beyond individual interactions and reflected broader processes of professional socialization. Participants described learning implicit expectations regarding loyalty, silence, and professional allegiance through repeated observations of everyday clinical practice. These hidden organizational norms influenced how learners understood their relationships with colleagues, patients, and the medical profession itself.

*“From the moment he stepped into medical school, he was taught not to challenge his professors and to remain silent…. The hierarchy of seniority that he had learned over the years had formed in his mind … it was ‘us’ (physicians) against ‘them’ (patients).” (EG1AY)*

Across narratives, hierarchy influenced PIF not merely by defining formal authority but by shaping learners’ opportunities to participate, question, and develop professional agency. Supportive leadership practices that encouraged collaboration and psychological safety promoted active engagement and identity development, whereas rigid hierarchical cultures reinforced conformity and constrained learners’ capacity to negotiate their emerging professional identities.

#### 3.2.3. Societal and systemic influences

Beyond immediate clinical learning environments, participants described broader healthcare and societal conditions as important contextual influences on PIF. Rather than operating independently, institutional priorities, service demands, and societal expectations shaped the learning opportunities available to students and influenced how they interpreted the professional role of physicians.

Participants consistently described societal expectations as contributing to an intensified sense of professional responsibility. Physicians were perceived as occupying a position associated with exceptionally high public expectations, where clinical competence was often assumed to be infallible. Learners described this expectation as generating emotional pressure while simultaneously reinforcing their awareness of professional accountability.

*“People often believe that doctors do not make mistakes. When a mistake happens, trust collapses immediately. But we are human too, and carrying that expectation creates a heavy pressure.” (F1AY)*

Participants also described healthcare systems increasingly organized around service delivery, where heavy clinical workloads frequently reduced opportunities for teaching, dialogue, and reflective learning. Under these conditions, clinical education often became task-oriented, limiting students’ opportunities to understand the reasoning underlying clinical decisions and reducing meaningful participation in patient care.

*“When the ward is busy, residents usually give quick instructions like ‘Do this, do that.’ As students, we try to keep up, but there is often no time to explain the reasoning behind what we are asked to do.” (B5AY)*

Rather than affecting learning alone, these systemic conditions influenced how learners understood the medical profession itself. Participants described recognizing tensions between the ideals promoted within formal medical education and the realities of contemporary clinical practice, where service demands frequently competed with educational responsibilities. These experiences shaped not only what students learned but also how they interpreted professional responsibility, clinical decision-making, and their future roles as physicians.

Across narratives, societal expectations and healthcare system constraints functioned as contextual conditions that indirectly shaped professional identity formation by influencing the quality of clinical learning, opportunities for reflective dialogue, and learners’ participation in authentic professional practice.

### 3.3. Conceptual Framework of Professional Identity Formation

The integrated analysis of reflective narratives and interviews resulted in the development of a conceptual framework illustrating how PIF emerges through the dynamic interaction of learners, professional communities, and multiple contextual influences (Figure 1). Rather than depicting identity formation as a linear developmental process, the framework conceptualizes PIF as a reciprocal and iterative process occurring within interconnected educational and clinical environments.

**Figure 1:**
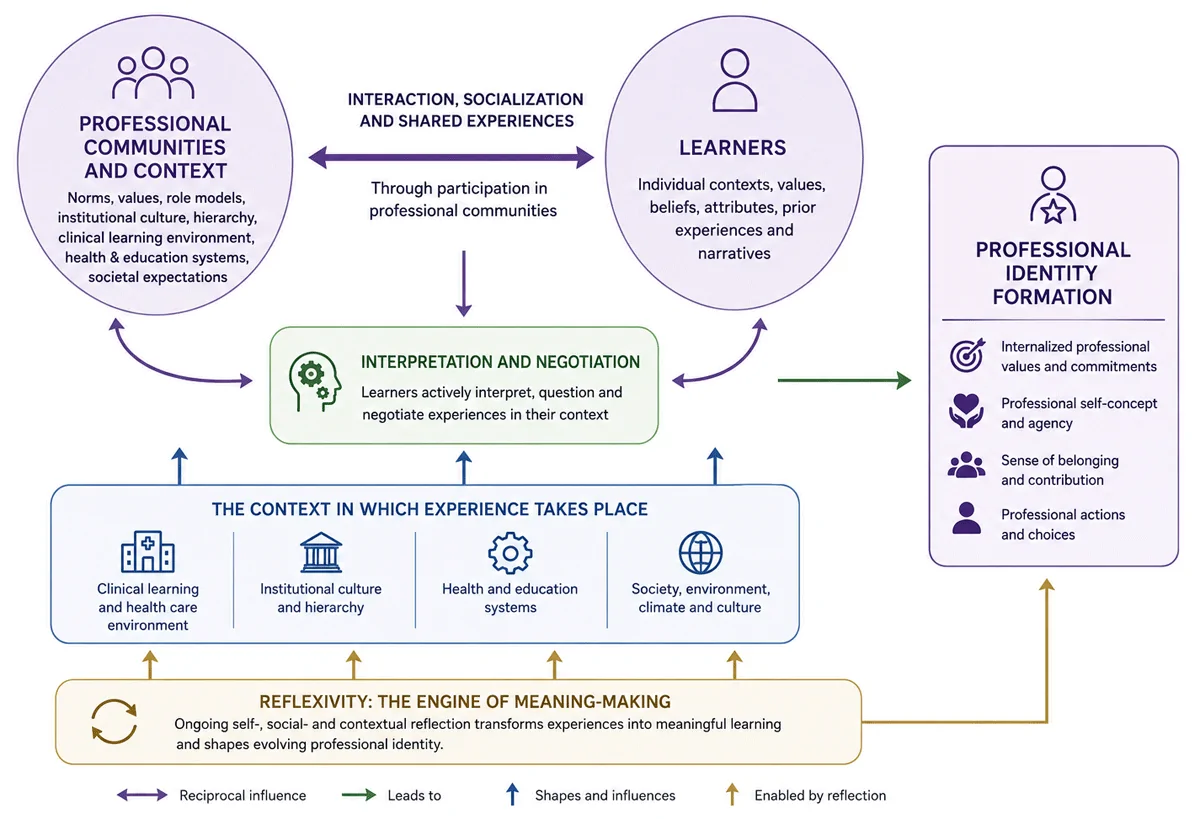
Conceptual framework illustrating how contextual experiences are interpreted and integrated into professional identity formation.

Three interrelated contextual domains were identified as shaping professional identity formation:

1. **Clinical learning and healthcare environments**, including organizational culture, participation, supervision, and team interactions;
2. **Societal and cultural contexts**, encompassing public expectations, professional values, and sociocultural norms;
3. **Healthcare and educational systems**, including institutional structures, service demands, and curricular influences.

Across these domains, learners’ individual characteristics, prior experiences, and modes of engagement functioned as mediating factors that influenced how clinical experiences were interpreted and incorporated into their developing professional identities. Reflective meaning-making emerged as a continuous process through which learners integrated contextual experiences, negotiated professional values, and reconstructed their understanding of what it means to become a physician.

Overall, the framework suggests that professional identity formation is shaped not solely through formal educational activities but through the continuous interaction between learners, professional communities, and the broader clinical, organizational, and sociocultural contexts in which learning occurs (Figure 1).

## 4. Discussion

In relation to the research questions, three overarching findings emerged. First, PIF was framed narratively as an ongoing relational and moral negotiation shaped through clinical encounters and role modelling. Second, contextual forces—spanning clinical learning environments, hierarchical cultures, and broader societal and systemic structures—significantly influenced how professional roles were interpreted and enacted. Third, reflection and emotional processing functioned as critical yet inconsistently supported mechanisms through which clinical experiences were integrated into developing professional identities.

Consistent with contemporary PIF frameworks [6], our findings confirm that professional identity develops through participation, social interaction, and authentic clinical experiences. However, our findings further suggest that contextual influences do not shape professional identity directly. Rather, learners actively construct professional identity by assigning personal and professional meaning to authentic clinical experiences, role-model behaviours, and organizational tensions. This interpretive process emerged as a central mechanism linking context with identity development throughout the narratives.

The clinical learning environment emerged as a central determinant of identity development. Supportive contexts characterized by mentorship, dialogical engagement, and constructive feedback fostered belonging and professional alignment, consistent with prior evidence linking positive learning climates to professional growth [20,21]. Conversely, service-dominant structures and workload pressures constrained participation and reflective engagement. While similar contextual tensions have been widely documented, our findings suggest that these influences are not simply experienced as external constraints. Rather, learners actively interpret, negotiate, and integrate such experiences into their developing professional identities through ongoing narrative meaning-making. Role modelling functioned as a mediating mechanism within these contexts. In environments where structured teaching was limited, learners relied heavily on observing clinicians’ relational conduct, ethical positioning, and emotional regulation. This finding aligns with research emphasizing workplace learning and mentorship in identity development [22]. Importantly, the findings underscore the potency of the hidden curriculum: implicit norms embedded in everyday clinical interactions often exerted stronger influence on identity formation than formally articulated competency frameworks. Hierarchical cultures, in particular, constrained questioning and ethical dialogue, echoing evidence that rigid hierarchies suppress learner agency [23,24]. However, our findings further illustrate how such hierarchies shape not only participation but learners’ relational positioning—sometimes fostering an “us versus them” orientation toward patients.

Beyond institutional settings, societal and systemic dynamics also shaped professional identity formation. Public expectations of physicians as infallible figures imposed psychological pressure, influencing learners’ self-perception and patient interactions [25]. System-level constraints, including high patient volumes and limited dialogical teaching, were perceived as privileging technical efficiency over relational care, consistent with concerns about mechanistic tendencies in contemporary medical education [26]. By foregrounding these tensions narratively, this study demonstrates that PIF is not merely an educational trajectory but a socially embedded and structurally mediated process. Notably, variations across clinical and regional contexts suggest that even within a shared national framework, professional identity may be differentially constructed depending on institutional culture and sociocultural climate.

Across narratives, the “good physician” was not primarily framed as technically proficient, but as ethically anchored, relationally attentive, emotionally regulated yet responsive, reflective, and contextually aware. Competence remained essential; however, learners consistently emphasized principled conduct, dialogical communication, and moral accountability as defining characteristics of professional identity. This framing aligns with scholarship suggesting that competency-based models alone are insufficient to capture the relational, interpretive, and socially constructed dimensions of physicianhood [13,14,15]. By emphasizing relational and contextual dimensions, these findings extend critiques of purely performance-driven conceptions of professionalism.

Taken together, the findings suggest several implications for medical education. Embedding brief structured reflective pauses into ward rounds, facilitating narrative debriefings following emotionally charged encounters, and explicitly addressing hierarchical communication norms may strengthen learners’ ethical positioning and relational awareness. Faculty development initiatives that strengthen educators’ dual roles as clinicians and mentors may help bridge this gap [21]. Faculty development should move beyond technical supervision to include mentorship training that sensitizes clinicians to hidden curriculum dynamics and relational modelling. Additionally, incorporating humanities and social sciences into curricula may support learners in navigating ethical tensions and societal expectations [27]. Gradual assumption of clinical responsibility, supported by supervision and feedback, appears critical for sustaining humanistic and context-sensitive professional development [28].

These findings may be translated into practice through brief 15–20-minute narrative debriefings following ward rounds or case discussions, longitudinal reflective writing integrated into clinical clerkships, and faculty development programs emphasizing dialogic feedback, role modelling, and recognition of hidden curriculum dynamics. The feasibility of these approaches, however, should be considered within the realities of clinical education. Heavy workloads, competing service priorities, limited faculty time, and hierarchical organizational cultures may constrain opportunities for structured reflection and mentorship. Embedding these activities within existing clinical workflows, rather than introducing additional stand-alone sessions, may therefore provide a more feasible and sustainable approach while minimizing additional demands on clinical teaching.

Across the findings, PIF can be understood within a socio-cultural paradigm informed by third-generation activity theory. Identity development emerged through learners’ participation in multiple interacting activity systems, including clinical environments, educational structures, and broader healthcare contexts [11]. The tensions described by participants—such as service demands versus learning opportunities, hierarchy versus learner agency, and technical efficiency versus humanistic care—reflect the systemic contradictions central to activity-theoretical analysis. However, the present findings extend activity-theoretical interpretations by identifying narrative integration and contextual reflexivity as key mediating mechanisms. While activity theory conceptualizes identity as shaped through participation in collective practices, our results suggest that identity development also depends on how learners narratively interpret and emotionally process their situated experiences. PIF therefore appears as a relational, contextually mediated, and narratively constructed process rather than a linear developmental trajectory.

Our findings closely illustrate several core constructs of third-generation activity theory [12]. Supportive clinical environments represented enabling community structures, whereas hierarchical communication norms reflected institutional rules that shaped learners’ agency. Similarly, tensions between service provision and educational priorities exemplified contradictions between interacting activity systems. Rather than viewing these contradictions solely as barriers, participants interpreted them narratively as opportunities for negotiating professional identity.

### 4.1. Strengths and Limitations

A major strength of this study is its multicenter, narrative-based, and contextual design, incorporating perspectives from learners and role models across accredited public and private medical schools from all geographic regions of Türkiye. By presenting data from a distinct cultural context, the study contributes a valuable cross-cultural perspective to the PIF literature. However, the first year of data collection coincided with the COVID-19 pandemic, limiting clinical interactions and socialization opportunities. Additionally, private medical schools were underrepresented due to inclusion criteria constraints. Although the inclusion of diverse institutional contexts strengthens the analytical transferability of the findings, the study was conducted within the specific cultural, educational, and healthcare context of Türkiye. Therefore, the findings should be interpreted in relation to local institutional cultures, healthcare structures, and professional norms, and their applicability to other settings should be considered through contextual nature rather than assuming generalization.

## 5. Conclusion

This multicenter narrative study demonstrates that professional identity formation is shaped not simply by exposure to clinical environments, role models, and systemic influences, but through how learners actively interpret, negotiate, and integrate these experiences into their developing professional identities. Clinical, institutional, healthcare-system, and sociocultural contexts interacted to shape learners’ participation, relationships, agency, and understanding of what it means to become a physician. Role modelling similarly emerged not as a process of passive imitation, but as one in which observed professional behaviours were critically interpreted, accepted, adapted, or rejected. The proposed conceptual framework therefore positions PIF as a dynamic and relational process arising from the interplay between contextual influences, social interactions, and learners’ narrative meaning-making. By identifying interpretation and contextual reflexivity as mechanisms through which experiences and systemic tensions become professionally meaningful, this study extends socio-cultural understandings of PIF and highlights the need for learning environments that support participation, dialogue, mentorship, and reflective meaning-making.

## Data Accessibility Statement

The data supporting the findings of this study are available from the corresponding author upon reasonable request, in accordance with ethical guidelines.

## Additional File

The additional file for this article can be found as follows:

10.5334/pme.2658.s1 Supplementary Material 1. The questions used in semi-structured in-depth interviews.

## References

[1] Cruess RL, Cruess SR, Steinert Y. Teaching medical professionalism. New York: Cambridge University Press; 2016. DOI: 10.1017/CBO9781316178485

[2] Jarvis-Selinger S, Pratt DD, Regehr G. Competency is not enough: integrating identity formation into the medical education discourse. Acad Med. 2012;87(9):1185–1190. DOI: 10.1097/ACM.0b013e3182604968 22836834

[3] Cruess RL, Cruess SR. Expectations and obligations: professionalism and medicine’s social contract with society. Perspect Biol Med. 2008;51(4):579–598. DOI: 10.1353/pbm.0.0045 18997360

[4] Leo T, Eagen K. Professionalism education: the medical student response. Perspect Biol Med. 2008;51(4):508–516. DOI: 10.1353/pbm.0.0058 18997353

[5] Finn G, Gardner J, Sawdon M. “You are judged all the time!” Students’ views on professionalism: a multicenter study. Med Educ. 2010;44(8):814–825. DOI: 10.1111/j.1365-2923.2010.03743.x 20633221

[6] Sarraf-Yazdi S, Pisupati A, Goh CK, Ong YT, Toh YR, Goh SPL, Krishna LKR. A scoping review and theory-informed conceptual model of professional identity formation in medical education. Med Educ. 2024;58(10):1151–1165. DOI: 10.1111/medu.15399 38597258

[7] Sternszus R, Steinert Y, Razack S, Boudreau JD, Snell L, Cruess RL. Being, becoming, and belonging: Reconceptualizing professional identity formation in medicine. Front Med (Lausanne). 2024;11:1438082. DOI: 10.3389/fmed.2024.1438082 PMC11383779 39257893

[8] Gülpınar MA. In search of a new paradigm. World Med Educ. 2021;20(60):82–100. DOI: 10.25282/ted.772542

[9] Bruner J. A narrative model of self-construction. Ann N Y Acad Sci. 1997;818(1):145–161. DOI: 10.1111/j.1749-6632.1997.tb48253.x

[10] Frolli A, Cerciello F, Ciotola S, et al. Narrative approach and mentalization. Behav Sci (Basel). 2023;13(12):994. DOI: 10.3390/bs13120994 PMC10740439 38131850

[11] Engeström Y. Activity theory and individual and social transformation. In: Engeström Y, Miettinen R, Punamäki RL, editors. Perspectives on activity theory. Cambridge: Cambridge University Press; 1999. DOI: 10.1017/CBO9780511812774

[12] Bakhurst D. Reflections on activity theory. Educ Rev. 2009;61(2):197–210. DOI: 10.1080/00131910902846916

[13] Jarvis-Selinger S, MacNeil KA, Costello GRL, et al. Understanding professional identity formation in early clerkship: a novel framework. Acad Med. 2019;94(10):1574–1580. DOI: 10.1097/ACM.0000000000002835 31192797

[14] Leep Hunderfund AN, Kumbamu A, O’Brien BC, et al. “Finding my piece in that puzzle”: a qualitative study exploring how medical students envision their future professional identity. Acad Med. 2022. DOI: 10.1097/ACM.0000000000004799 35797546

[15] Pach J, Stoffels M, Schoonmade L, et al. The impact of educational activities on professional identity formation: a scoping review. Educ Res Rev. 2025;100704. DOI: 10.1016/j.edurev.2025.100704

[16] Shapiro J, Kasman D, Shafer A. Words and wards: a model of reflective writing and its uses in medical education. J Med Humanit. 2006;27:231–244. DOI: 10.1007/s10912-006-9020-y 17001529

[17] Hampton M. Reflective writing: a basic introduction. Available from: https://capstone.unst.pdx.edu/sites/default/files/Reflective-writing---a-basic-intro_0.pdf (accessed 10 Aug 2021).

[18] Creswell JW. *Eğitim araştırmaları: nicel ve nitel araştırmanın planlanması, yürütülmesi ve değerlendirilmesi* [Educational research: Planning, conducting, and evaluating quantitative and qualitative research]. 3rd ed. Istanbul, Türkiye: EDAM Yayınları; 2020.

[19] Gülpınar MA. A model proposal for qualitative data analysis, interpretation, and reporting: contextuality, reflectivity and narrativity. Prim Health Care Res Dev. 2024;25:e55. DOI: 10.1017/S1463423624000562 PMC11569848 39465640

[20] Fantaye AW, Kitto S, Hendry P, et al. Attributes of excellent clinician teachers and barriers to recognizing and rewarding clinician teachers’ performances and achievements: a narrative review. Can Med Educ J. 2022;13(2):57–72. DOI: 10.36834/cmej.73241 PMC9099178 35572019

[21] Huang J, Huang C, Chen J, et al. Perception of clinical teachers about their roles and current practice at affiliated hospitals of medical universities in China. Med Educ Online. 2024;29(1):2325182. DOI: 10.1080/10872981.2024.2325182 PMC10930135 38465652

[22] Leach H, Coleman JJ. William Osler in medical education. Postgrad Med J. 2019;95(1130):642–646. DOI: 10.1136/postgradmedj-2018-135890 31754055

[23] Tarrant C, Leslie M, Bion J, et al. A qualitative study of speaking out about patient safety concerns in intensive care units. Soc Sci Med. 2017;193:8–15. DOI: 10.1016/j.socscimed.2017.09.036 PMC5669358 28987982

[24] Keshmiri F, Raadabadi M. Perception and engagement in unprofessional behaviors of medical students and residents: a mixed-method study. BMC Prim Care. 2023;24:191. DOI: 10.1186/s12875-023-02153-y PMC10506320 37723441

[25] Gray K, Wegner DM. Moral typecasting: divergent perceptions of moral agents and moral patients. J Pers Soc Psychol. 2009;96(3):505–520. DOI: 10.1037/a0013748 19254100

[26] Serodio A, Kopelman BI, Bataglia R. The promotion of medical students’ moral development. Int J Ethics Educ. 2016;1(1):81–89. DOI: 10.1007/s40889-016-0009-8

[27] Desai MK, Kapadia JD. Medical professionalism and ethics. J Pharmacol Pharmacother. 2022;13(2). DOI: 10.1177/0976500X221111448

[28] Mak-van der Vossen M, Teherani A, van Mook W, et al. How to identify, address and report students’ unprofessional behaviour in medical school. Med Teach. 2020;42(4):372–379. DOI: 10.1080/0142159X.2019.1692130 31880194

