# Supplementary material for: Professional Identity Formation in Clinical Medical Education: A Multicenter Narrative Study of Contextual Mediation and Reflective Meaning-Making
Source: Perspect Med Educ. 2026 Nov 4;15(1):1159–73. doi: 10.5334/pme.2658 (PMC13741948; doi:10.5334/pme.2658)
Supplement: Supplementary file 1 [file pme-15-1-2658-s1.pdf]

Supplemental material 1. The questions used in semi-structured in-depth interviews

Semi-Structured In-Depth Interview Questions with Students

1. What moments or experiences do you think have been influential in shaping your professional identity? Could you narrate these experiences?
2. Who is the medical educator(s) you consider as a role model and who has positively influenced your professional identity formation during your clinical education? Why? Could you share one or two memorable experiences with this person?
3. Have you had any negative experiences during your clinical training that made you say, *"I will not be a physician like this"*? Without specifying any identities, could you narrate one or two such experiences, explaining what happened, how it happened, and how it affected you?

Semi-Structured In-Depth Interview Questions with Role Models

- How would you describe your professional identity and medical identity in your own words? Could you discuss the factors that have influenced the formation of your professional identity?
- How would you describe the medical identity of the role models who influenced your own professional identity formation? Could you share one or two memorable experiences with these individuals?
- As a clinician and educator, what recommendations do you have regarding the professional identity formation process in clinical education?
